# What do we know about IL-6 in COVID-19 so far?

**DOI:** 10.52601/bpr.2021.200024

**Published:** 2021-06-30

**Authors:** Jingrui Jiang, Jun Wang, Lulu Yao, Shenghan Lai, Xueji Zhang

**Affiliations:** 1 Tongji Medical College, Huazhong University of Science and Technology, Wuhan 430030, China; 2 Department of Biomedicine and Biopharmacology, Hubei University of Technology, Wuhan 430068, China; 3 National 111 Center for Cellular Regulation and Molecular Pharmaceutics, Wuhan 430068, China; 4 Department of Pathology, Johns Hopkins University School of Medicine, MD 21287, USA; 5 Guangdong Laboratory of Artificial Intelligence and Digital Economy (SZ), School of Biomedical Engineering, Shenzhen University, Shenzhen 518037, Guangdong, China

**Keywords:** IL-6, IL-6R, Cytokine storm, COVID-19, Monoclonal antibody

## Abstract

Interleukin 6 (IL-6) is a cytokine with dual functions of pro-inflammation and anti-inflammation. It is mainly produced by mononuclear macrophages, Th2 cells, vascular endothelial cells and fibroblasts. IL-6 binds to glycoprotein 130 and one of these two receptors, membrane-bound IL-6R or soluble IL-6R, forming hexamer (IL-6/IL-6R/gp130), which then activates different signaling pathways (classical pathway, trans-signaling pathway) to exert dual immune-modulatory effects of anti-inflammation or pro-inflammation. Abnormal levels of IL-6 can cause multiple pathological reactions, including cytokine storm. Related clinical studies have found that IL-6 levels in severe COVID-19 patients were much higher than in healthy population. A large number of studies have shown that IL-6 can trigger a downstream cytokine storm in patients with COVID-19, resulting in lung damages, aggravating clinical symptoms and developing excessive inflammation and acute respiratory distress syndrome (ARDS). Monoclonal antibodies against IL-6 or IL-6R, such as tocilizumab, sarilumab, siltuximab and olokizumab may serve as therapeutic options for COVID-19 infection.

## INTRODUCTION

In the past two decades, severe acute respiratory syndrome coronavirus (SARS-CoV) and Middle East respiratory syndrome coronavirus (MERS-CoV) had been passed from animal to human, causing severe respiratory diseases SARS and MERS worldwide. In December 2019, another coronavirus SARS-CoV-2 was found in patients with later-called COVID-19 diseases in Wuhan City, Hubei Province, China, which is also transmitted from human to human. This virus spreads rapidly throughout China after its identification, and then around the world, though the origin of SARS-CoV-2 still stays as a puzzle. As of February 22, 2021, there were more than 111,697,446 COVID-19 patients and more than 2,473,085 deaths worldwide.

SARS-CoV-2 belongs to β-coronaviruses, as SARS-CoV and MERS-CoV do (Dai *et al*. 2020). It has been noted that clinical features of COVID-19 infection were similar to those of SARS and MERS (Assiri *et al*. 2013). For example, respiratory epithelial cells are the first target infected by these viruses, which all cause diffuse interstitial pulmonary fibrosis and acute respiratory failure (Yin and Wunderink 2018). However, very few patients with COVID-19 infection had obvious upper respiratory symptoms, such as nosebleeds, sneezing or sore throat. It indicates that infections by COVID-19 may occur in the lower respiratory tract, instead of the upper respiratory tract. This is different from SARS infection, which occurred at mouth and intestines (Wang 2004). Furthermore, unlike diarrhea symptoms found in about 20%–25% of patients with MERS or SARS, COVID-19 patients rarely developed intestinal disorders (Eastin and Eastin 2020a). Moreover, the fatality rate of COVID-19 is around 2.3%, lower than the mortality rate of SARS (9.5%) and much lower than that of MERS (34.4%) (Petrosillo *et al*. 2020). The mortality rates of SARS, MERS and COVID-19 were higher than those caused by other coronaviruses (Magro 2020). Similar to pathogenesis of SARS and MERS, COVID-19 also stimulates primary inflammatory response and further secondary inflammatory response in its host, which eventually results in organ failure and even death of patients (Qiao and Dong 2020).

Studies have shown that COVID-19 infection may induce upregulation of interleukin 6 (IL-6), interleukin 16 (IL-16), interleukin 12 (IL-12), tumor necrosis factor-α (TNF-α), and a series of downstream cytokine cascade reactions (Mehta *et al*. 2020). It has been proved that viral infection induces IL-6 production through TNF-α (Elias and Lentz 1990; Kurokouchi *et al*. 1998; Tseng *et al*. 2010). Downstream IL-6 activation has been suggested to be a turning point when initial COVID-19 infection deteriorates into excessive inflammation and ARDS (Lipworth *et al*. 2020). IL-6, as well as its physiological receptors, is therefore considered as a promising therapeutic target in critically ill patients infected by COVID-19 (Gubernatorova *et al*. 2020). The purpose of this review is to discuss the potential mechanism of IL-6 during cytokine storms in COVID-19 patients from the perspective of its structure, general function and what we have known about it in COVID-19 pandemic so far. In addition, most recent progress on inhibitors of IL-6 or its receptors, tocilizumab, sarilumab, siltuximab and olokizumab, is also summarized from perspective of clinical trials for COVID-19 therapy.

## STRUCTURE AND GENERAL FUNCTION OF IL-6

**Figure 1:**
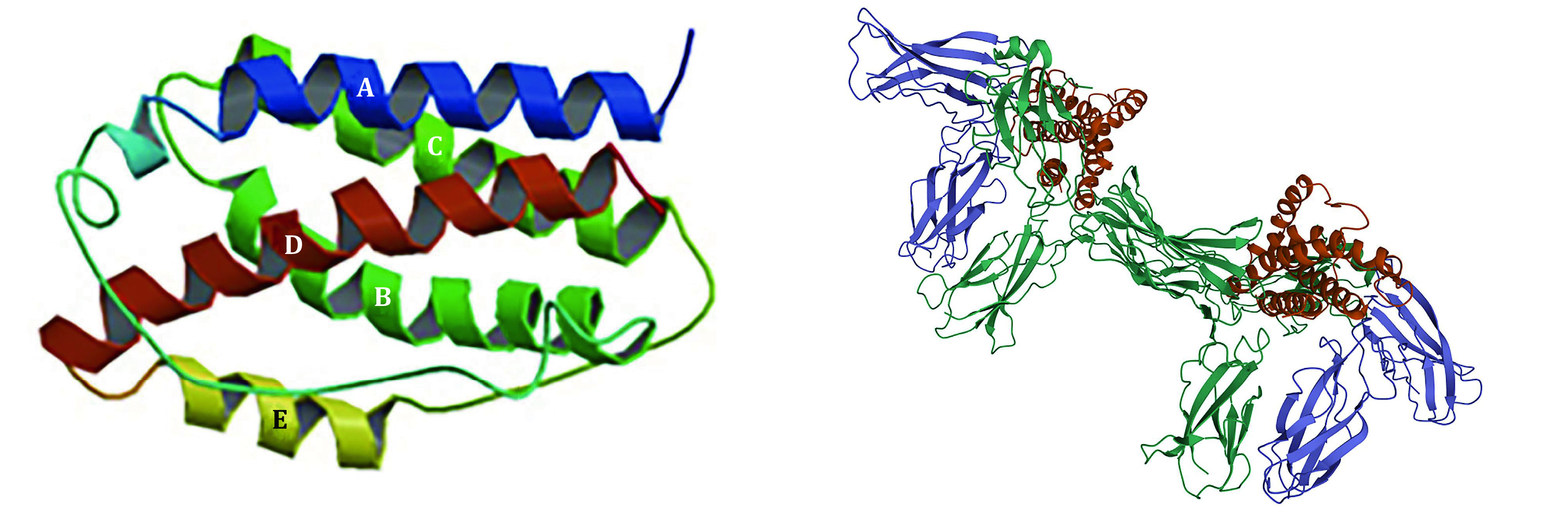
The hexamer of IL-6 band structure (DOI: 10.2210/pdb1IL6/pdb) (left), and hexamer (IL-6/IL-6R/gp130) (DOI: 10.2210/pdb1P9M/pdb) (right)

IL-6 is a single chain liposoluble phosphorylated glycoprotein. It has a spiral structure, composed of four helix bundles (A–D). Helix A and B extend in one direction, while C and D run in the opposite direction (Fig. 1). Helix E is a short chain between helix C and D, located outside the main helix bundles (Kaur *et al*. 2020). IL-6 is composed of 212 amino acids, with the theoretical isoelectric point (pI) of 6.71. It contains six Ser, two Thr, and one Tyr, which are sites for phosphorylation by protein kinase. Amino acids 11–33 form the low complexity region (LCR). Amino acids 30–56 are dominant antigen epitopes. Amino acids 57–210 are structurally conservative (Yu *et al*. 2016).

Due to its unique structure, IL-6 has multiple biological functions (McGonagle *et al*. 2020). IL-6 signals by binding to type I receptor complex on cell membrane, which consists of one ligand-binding glycoprotein, IL-6 receptor (IL-6Rα), and the signal-transducing component gp130. The coding region of IL-6R cDNA is 1.4 kb in length, encoding 468 amino acids. IL-6Rα is an alpha chain with the molecular weight of 80 kDa, also known as CD126 (Varghese *et al*. 2002; Zohlnhöfer *et al*. 1992). There are six potential sites in IL-6R for N-glycosylation. Five of them are located outside cell, and the sixth one is located in cytoplasm (Simpson *et al*. 1997). IL-6R has two forms, namely membrane-bound IL-6R (mIL-6R) and soluble IL-6R (sIL-6R). IL-6R is mainly expressed in liver cells, neutrophils, monocytes and T cells. It helps execute physiological functions mediated by IL-6.

**Figure 2:**
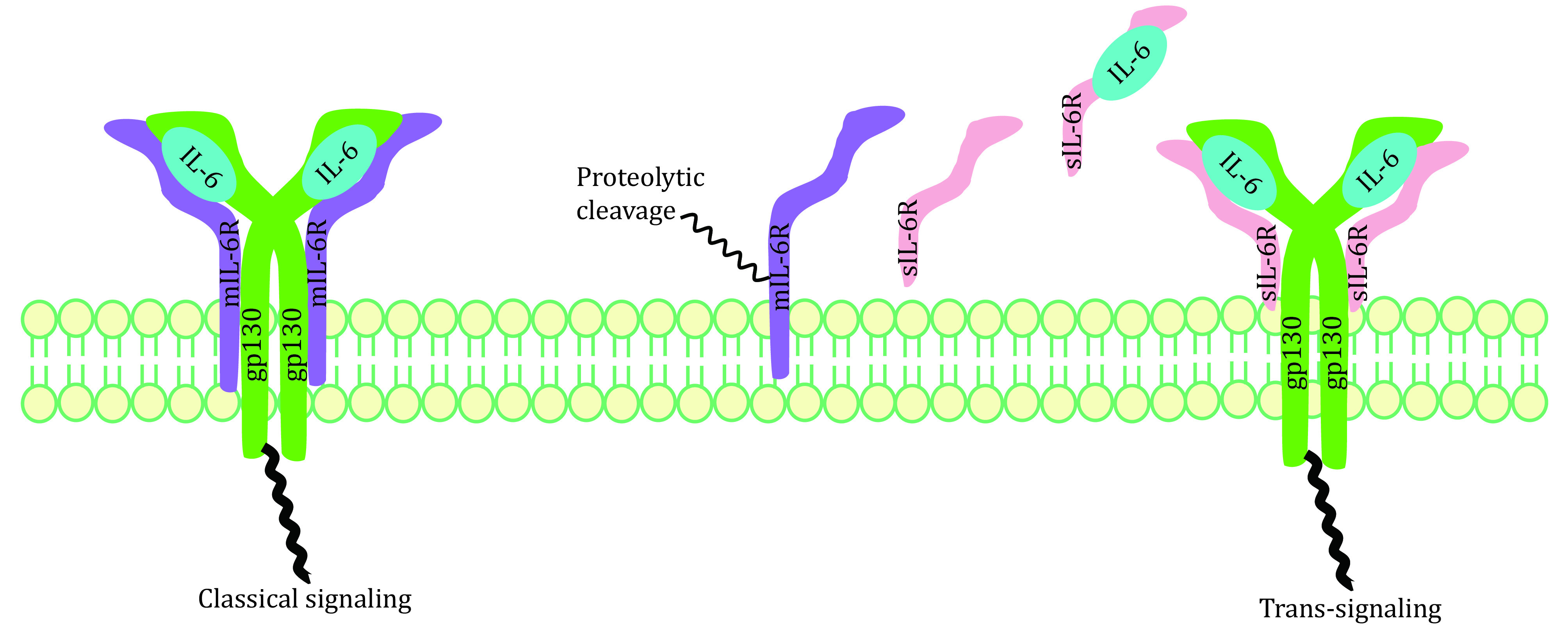
Two IL-6 signaling pathways: classical signaling and trans-signaling

Whether IL-6 exerts pro-inflammatory or anti-inflammatory effects mainly depends on its receptor. mIL-6R mediates anti-inflammation, which is the classical signaling pathway occurring mainly in leukocytes and hepatocytes. Pro-inflammation or the trans-signaling pathway is activated by IL-6/sIL-6R complex, which is crucial for lymphocyte trafficking into the inflamed area via controlled chemokine expression (Rose-John and Heinrich 1994; Rose-John *et al*. 2007). Many non-immune cells, including stromal and epithelial cells, also exhibit inflammatory responses (Jones *et al*. 2005). Whether it is pro-inflammatory or anti-inflammatory, the extracellular part of IL-6R is always responsible for IL-6 binding. It first binds to IL-6R via its CBD domain and then attaches to gp130, leading to formation of IL-6/IL-6Rα/gp130 complex and homodimerization of gp130. Afterwards, the ras/raf/Mitogen-activated protein (MAP) kinase (MAPK) pathway gets activated. Signal transducer and activator of transcription factors are recruited, followed by phosphorylation and dimerization whereupon they translocate into the nucleus and activate target genes (Scheller *et al*. 2011). Different biological effects would follow thereafter, depending on the type of receptor (Fig. 2).

Excessive sIL-6R has been suggested to correlate with cytokine storm. Two mechanisms have been reported for generation of sIL-6R (Assiri *et al*. 2013; Lust *et al*. 1992): (1) transcription of an alternative spliced IL-6R-mRNA lacking the transmembrane and cytosolic domains (Lust *et al*. 1992); (2) proteolysis of mIL-6R dependent on a metalloprotease activity (Düsterhöft *et al*. 2019; Foldvari-Nagy *et al*. 2021; Garbers *et al*. 2011; Müllberg *et al*. 1993; Vollmer *et al*. 1996). In one mouse model study of acute inflammation, apoptosis-induced shedding of IL-6R by neutrophils promoted formation of IL-6/sIL-6R complexes, therefore conducting IL-6 trans-signaling in endothelial cells (Lust *et al*. 1992; Scheller *et al*. 2011). Then these cells were stimulated to secrete chemokines for gathering of mononuclear phagocytic cells. Application of neutralizing antibodies against IL-6 or soluble gp130-Fc in this model led to normal influx of neutrophils but to damaged monocytes influx into the inflamed area. Thus, IL-6R shedding may represent important steps for the resolution of inflammatory responses. Elevated sIL-6R breaks the IL-6-mediated anti-inflammatory and pro-inflammatory balance, causing cytokine storms in the end.

IL-6 is a Janus-face cytokine (Jones and Jenkins 2018). It can coordinate with other cytokines to control cell proliferation and differentiation, angiogenesis, as well as regulation of immune and inflammatory responses (Matsuda and Kishimoto 1998; McGonagle *et al*. 2020; Tisoncik *et al*. 2012; Zong *et al*. 2020). IL-6 regulates host defense capabilities through various immune stimulation mechanisms: mediating monocyte differentiation into macrophages (Chomarat *et al*. 2000), regulating antigen-dependent B cell differentiation (Urashima *et al*. 1996), promoting B cell to produce IgG (Yang *et al*. 2016) and inhibiting Th1 polarization response to activate Th2 cells (Diehl and Rincón 2002). Researchers have studied IL-6 knock-out mice and found that IL-6 was critical during recovery from viral infections. However, although IL-6 is essential during up-regulation of T cell responses, tissue remodeling and repair, it also promotes viral persistence during antiviral immune responses (Velazquez-Salinas *et al*. 2019).

## THE ROLE OF IL-6 IN CYTOKINE STORM DURING COVID-19 PATHOGENESIS

### Cytokine storm in COVID-19 pathogenesis

Cytokine release syndrome (CRS) has been related to organ failures in severe COVID-19 patients (Berlin *et al*. 2020; Huang *et al*. 2020; Zhang *et al*. 2020b). CRS is characterized by high fever, thrombocytopenia, hyperferritinemia, as well as increase of inflammatory markers (such as C-reactive protein (CRP)) (Hashizume 2020). Cytokine storm syndrome (CSS) is synonymous with CRS, which is characterized by overwhelming systemic inflammation, hyperferritinaemia, haemodynamic instability and multiple organ failure (MOF) (Gao *et al*. 2021). Increasing evidences have showed the existence of CSS in critical ill COVID-19 patients.

In the lower respiratory tract, COVID-19 invaded type II alveolar epithelial (AE2) cells through ACE2 receptor. Generally, AE2 cells don’t participate in active gas exchange, but are critical to maintain integrity and function of alveoli (Li *et al*. 2020a). Destruction of AE2 cells and increase of cell permeability led to release of COVID-19 virus, which entered underlying tissues to advance infection or were captured by macrophages, dendritic cells, and neutrophils, causing the next-step propagation (Chen *et al*. 2020d). Necrotic cells got ruptured after being infected, and intracellular materials were released, triggering an inflammatory response in the body. COVID-19 virus also activated the innate immune system. Macrophages and other innate immune cells not only captured virus, but also released a large number of cytokines, including interleukin-1β (IL-1β), IL-6, IL-12, IL-16 and TNF-α (Wan *et al*. 2020), which promoted Th0 cells to differentiate and migrate to affected organs (Monteleone *et al*. 2020). Once inflammation in lung tissues was out of control, excessive immune response called gathering of a number of immune cells to accumulate in lung. At the same time, abnormally high level of cytokines, *i.e*. cytokine storm, in lung induced tremendous activation of these immune cells, which ultimately damaged pulmonary capillary endothelial cells and alveolar epithelial cells. Massive exudate from these damaged cells accumulated in lung mucus and blocks the airway, which in turn caused acute respiratory distress syndrome (ARDS), sepsis and organ failure. It has been generally accepted that a cytokine storm was responsible for deterioration of COVID-19 patients. Moreover, comorbidities may exacerbate clinical manifestations of COVID-19 patients, which include cardiovascular disease, diabetes, respiratory diseases, high blood pressure and being aged (Guo *et al*. 2020).

During viral infection, another mechanism that drives cytokine storm has been suggested to be increased vascular permeability, which led to infiltration of effector cells, producing more inflammatory molecules and aggravating overproduction of cytokines (Ye *et al*. 2020). In addition, leakage of blood vessels allowed virus to spread to other tissues and organs, impairing their functions. Subsequently, under the continuous action of cytokines, more and more inflammatory exudates and red blood cells entered alveoli, resulting in respiratory failure (Zhang *et al*. 2020a). The cytokine storm caused intensification of this new coronavirus pneumonia and ARDS. Eventually, cytokine storms in the lung caused systemic cytokine storms, resulting in systemic organ failure in COVID-19 patients and even death (Renu *et al*. 2020; Shimabukuro-Vornhagen *et al*. 2018; Zhang *et al*. 2020a). Other than COVID-9 patients, severe SARS and MERS patients also experienced various degrees of cytokine storms (Mahmudpour *et al*. 2020). Level of IL-6 in patients with severe SARS was higher than in healthy subjects (Herold *et al*. 2020b). Among these patients, IL-6 caused nonspecific reactions due to SARS invasion of the respiratory tract (Wang *et al*. 2004).

### IL-6 in cytokine storm of COVID-19

Factors of age, sex, life style and pathological conditions influence serum IL-6 concentrations, which lead to a wide variation of IL-6 levels in adults (Alende-Castro *et al*. 2020). However, significantly elevated IL-6 has been reported in population with inflammatory diseases, including SARS-CoV-2-induced disease (Alende-Castro *et al*. 2020; Ilmarinen *et al*. 2016).

Elevated IL-6 and nonspecific symptoms were observed in patients with COVID-19 (Ruan *et al*. 2020). Multiple studies have shown that there was a strong correlation between serum IL-6 levels and respiratory failure in patients with COVID-19 (Chen *et al*. 2020c; Coomes and Haghbayan 2020; Zhang *et al*. 2020a). For example, there existed a strong correlation between elevated IL-6 and the need for mechanical ventilation (*p* = 1.2 × 10^−5^). Additionally, the maximal IL-6 level (cutoff = 80 pg/mL) predicted respiratory failure with high accuracy (*p* = 1.7 × 10^−8^, AUC = 0.98), and patients with IL-6 levels higher than 80 pg/mL had 22 times higher odds of respiratory failure than those with normal IL-6 levels (Herold *et al*. 2020a). It suggests that IL-6 might be a key factor for clinicians to determine whether patients required mechanical ventilation support with a ventilator or not (Tisoncik *et al*. 2012). Another study for measurement of IL-6 in plasma showed that IL-6 levels elevated accompanying increased severity of illness in patients (healthy control: 0.8 ± 1.6 pg/mL; COVID_stable_: 45.9 ± 24.8 pg/mL; COVID_ICU_: 169.4 ± 70.7 pg/mL; *p* = 0.0001). IL-6 levels in the group of patients with severe community-acquired pneumonia (CAPICU group) (99.4 ± 40.5 pg/mL) were significantly higher than in COVID_stable_ patients (*p* = 0.0001), but significantly lower than in COVID_ICU_ patients (*p* = 0.0005) (McElvaney *et al*. 2020).

It seemed that rapidly elevated IL-6 broke the balance between anti-inflammation and pro-inflammation regulated by both adaptive and innate immune systems (Sharan Tripathi *et al*. 2010). Early pathologic studies on clinical specimens of COVID-19 patients revealed that patients had double-side diffuse alveolar damage (DAD) accompanied by cell mucinous fibroma exudate (Miossec and Kolls 2012). Subsequent flow cytometry analysis of peripheral blood revealed that CD4+ and CD8+ cells were reduced, but Th17 cells increased. Th17 cells are helper T cells. Unlike Th0 cells, Th17 cells were mainly stimulated by IL-6 and IL-23 to induce cell differentiation (Shimabukuro-Vornhagen *et al*. 2018). In one study of 41 patients with SARS-CoV-2 infections in China, critically ill patients admitted in ICU had significantly lower blood lymphocytes than those patients who didn’t need ICU care. Levels of CD4+ and CD8+ T cells in blood were diminished, with B cells unchanged (Chen *et al*. 2020a). This type of persistent lymphopenia was also observed in patients previously infected by SARS-CoV and MERS-CoV (He *et al*. 2005). T lymphocytes, particularly CD4+ T cells, were suggested to participate in pathogenesis and outcome of coronavirus infections (Chen *et al*. 2010; Lavillegrand *et al*. 2021; Zhao *et al*. 2014).

Researchers also found that decreased function of natural killer cell (NK cell) might lead to elevated IL-6 (Cunningham *et al*. 2020). IL-6 can inhibit expression of perforin and granzyme B, resulting in reduction of apoptosis of virally infected cells (Cifaldi *et al*. 2015). Patients with abnormal perforin content in the body were more likely to experience a cytokine storm (Soy *et al*. 2020). Severe patients exhibited systemic excessive inflammation characterized by macrophage activation syndrome (MAS) and cytokine storm, which is also known as secondary haemophagocytic lymphohistocytosis (sHLH) (McGonagle *et al*. 2020). There is a significant correlation between plasma inflammatory markers and COVID-19 severity. However, correlation does not imply causality. It is likely that virus replication drives inflammatory response and subsequent disease severity, the exacerbated inflammation being an inappropriate host response that requires correction. In short, IL-6 plays a key role in cytokine storm of COVID-19 pathogenesis (Zhang *et al*. 2020a). Ongoing immunotherapy trials will be helpful to confirm the pathogenic role of IL-6 in patients with SARS-CoV-2 infection (Lavillegrand *et al*. 2021).

## INHIBITORS OF IL-6

Many potential solutions have been proposed for the treatment of COVID-19, including glucocorticoid (Li *et al*. 2020b), ulinastatin (Li and Ma 2020; Yang *et al*. 2020), azithromycin (Cipriani *et al*. 2020; Gabriels *et al*. 2020; Kelleni 2020) and corticosteroids (Hasan *et al*. 2020; Tobaiqy *et al*. 2020), but controversial opinions exist. Research of IL-6 inducing peptides/epitopes may shine lights on COVID-19 vaccine development (Dhall *et al*. 2020). Studies showed that corticosteroids were effective in some patients with severe ARDS associated with COVID-19 (Goursaud *et al*. 2020; Zha *et al*. 2020). Several institutes have suggested using corticosteroids to alleviate uncontrolled inflammation caused by COVID-19. However, the World Health Organization (WHO) does not recommend the use of corticosteroids, for they may exacerbate COVID-19-related lung damages (Chen *et al*. 2020b) and delay viral clearance (Eastin and Eastin 2020b). There are still no reliable studies evaluating efficacy and safety of corticosteroids in treatment of COVID-19 patients (Russell *et al*. 2020).

So far, systemic medicine treatment for COVID-19 has not been approved yet. However, according to the fact that IL-6 plays a crucial role in cytokine storms and the latter dramatically worsen symptoms of COVID-19 patients, IL-6 and its receptors may be good therapeutic targets. Several studies (Chen *et al*. 2020b; Fang *et al*. 2020; Huang *et al*. 2020) shared the same findings: elevated IL-6 levels existed in biological fluids of COVID-19 patients, and IL-6 might serve as a predictive biomarker for severity of COVID-19 infection (Gao *et al*. 2020). A large retrospective cohort study found that IL-6 levels were associated with mortality in COVID-19 patients (Zhou *et al*. 2020).

According to characteristics of IL-6 and its receptors, a feasible solution is to pursue the application of IL-6 antibody or sIL-6R antibody (Chuan *et al*. 2020; Liu *et al*. 2020). IL-6 pathway blockers may have a therapeutic effect in moderate to severe patients (Corominas *et al*. 2020). Studies have found that IL-6R blockers not only suppress cytokine storms, but also help to treat the early stages of CRS. Here, we focused on four antibodies, tocilizumab (TCZ) (Alzghari and Acuna 2020; Jones *et al*. 2010), sarilumab, siltuximab and olokizumab, which are known to block the IL-6 signaling pathway. Siltuximab blocks the downstream effects of IL-6 through anti-IL-6 (LactMed 2019). Binding of human IL-6 to both soluble and membrane-bound IL-6 receptors is hindered by this drug, and consequently formation of the hexameric signaling complex with gp130 on cell surface was prohibited, as well as inactivation of the Janus kinase/signal transducer and transcription signaling pathway (Palanques-Pastor *et al*. 2020). Different from TCZ and sarilumab, which are directly against IL-6R and have a selective downstream effect against IL-6, olokizumab aims at IL-6 instead of its receptor and selectively blocks the final assembly of the signaling complex (Kaplon and Reichert 2021). Quite a few clinical trials are ongoing for evaluation of effectiveness and safety of these monoclonal antibodies (Table 1).

**Table 1:** List of clinical trials on monoclonal antibodies against IL-6 or IL-6R for COVID-19

Drug	Participants	Location	Design	Inclusion criteria	Primary outcome measures	NCT/Reference
TCZ	450	Global	A randomized, double-blind, placebo-controlled, multicenter study	Confirmed patients, SpO_2_ ≤ 93% or PaO_2_/FiO2 < 300 mmHg	Clinical status as per the 7-category ordinal scale at day 28	04320615/ Hoffmann-La Roche 2020
TCZ	500	Spain	A multicenter, open-label study	Confirmed severe/critical patients, ≤2 days of TCZ or its candidate	To calculate the time of intubation, oxygen therapy, non-invasive mechanical ventilation and mortality rate	04445272/ Fundacion SEIMC-GESIDA 2020
TCZ	332	US	A multicenter, randomized, controlled phase 2 study	Confirmed adults with fever (*T* ≥ 38 °C)	Time to recovery (time frame: 28 days)	04479358/ University of Chicago 2020a
TCZ	310	Malaysia	An open-label, randomized, cross-over interventional study	Symptomatic patients with progressive disease and cytokine storm (presence of clinical and radiological signs)	The proportion and mean days of mechanical ventilation (time frame: average of 6 months)	04345445/ University of Malaya 2020
TCZ	400	Naples	A multicenter, single-arm, open-label, phase 2 study	Confirmed patients, oxygen saturation at rest in ambient air ≤93% or requiring oxygen therapy or mechanical ventilation	Mortality (within 2 weeks / 1 month)	04317092/National Cancer Institute 2020
TCZ	32	US	A non-randomized, single-arm, open-label study	Confirmed adults with fever (*T* ≥ 38 °C) and lung infiltration	Clinical response: resolution of fever (*T* < 38 °C, within 24 h); Biochemical response: time to CRP*^a^* normalization and its rate	04331795/ University of Chicago 2020b
Sarilumab	30	Spain	A randomized, open-label, single-center, comparative study	Confirmed adults with interstitial pneumonia*^b^*	Mean change in clinical status assessment using the 7-point ordinal scale at day 7, duration of hospitalization and death	04357808/ Maria del Rosario Garcia de Vicua Pinedo 2020
Sarilumab	40	Italy	A monocentric, single-center, escalation dose open-label study	Confirmed adults (18–85) with severe interstitial pneumonia and increased levels of D-dimer (or ≥ 1000 ng/mL)	Proportion of improvement in respiratory function (time frame: 6 weeks)	04386239/ ASST Fatebenefratelli Sacco 2020
Sarilumab	1912	US	An adaptive phase 2/3, randomized, double-blind, placebo-controlled study	Confirmed adults	Percent change in CRP (patients with serum IL-6 level greater than the upper limit of normal), proportion of patients with at least 1-point improvement using the 7-point ordinal scale (patients receiving mechanical ventilation at baseline)	04315298/Pharmac-euticals 2020
Sarilumab	60	Italy	A one-arm, open label, multicentric phase 2 study	Confirmed hospitalized adults with specified disease or index*^c^*	Change in a severity rating on a 7-point ordinal scale (time frame: 15 days)	04661527/Clinica Universidad de Navarra 2020
Sarilumab	120	US	A randomized, controlled, open label study	Confirmed veterans	Intubation or death (time frame: within 14 days)	04359901/Westyn Branch-Elliman 2020
Siltuximab	200	Spain	A randomized, open-label, phase 2 study	Confirmed hospitalized adults (≥5 days) with a maximum O_2_ support of 35%	Proportion of patients requiring ICU admission (time frame: 29 days)	04329650/Judit Pich Martínez 2020
Siltuximab	220	Italy	A retrospective study	Confirmed adults needing non-invasive or invasive ventilation	Mortality (time frame: 30 days)	04322188/Giuseppe Gritti 2020
Olokizumab	372	France	A multicenter, randomized, double-blind, adaptive placebo-controlled study	Confirmed adults with specified disease*^d^*	Proportion in response to the study therapy	04380519/R-Pharm International 2020
*^a^* CRP: C-reactive protein. *^b^* Interstitial pneumonia requiring admission and at least two of the following: 1. Fever ≥ 37.8 °C (tympanic); 2. IL-6 in serum ≥ 25 ng/mL (in the absence of a previous dose of prednisone or equivalent > 1 mg/kg) or PCR > 5 mg/dL; 3. Lymphocytes < 600 mm^3^; 4. Ferritin > 300 mcg/L that doubles in 24 h; 5. Ferritin > 600 mcg/L in the first determination and LDH > 250 U/L; 6. D-dimer (> 1 mg/L). *^c^* The patients with illness of any duration, with evidence of pneumonia, and severe disease as defined by at least one of the following: 1. High oxygen requirements (face mask with reservoir, non-invasive mechanical ventilation or high flow nasal cannula); 2. Lymphocytes < 0.8 × 10^9^ L^−1^; 3. Serum ferritin > 300 ng/mL; 4. Increased levels of D-dimer (> 1500 ng/mL) or D-dimer progressively increasing (over three consecutive measurements) and reaching ≥1000 ng/mL; 5. CPR > 10 mg/dL, or increasing over 24 h. *^d^* Having either of the following COVID-associated respiratory syndromes: 1. Pneumonia with oxygenation parameters SpO_2_ ≤ 93% (on room air) or respiratory rate greater than 30 min^−1^; 2. ARDS ( PaO_2_/FiO_2_ ≤ 300 mmHg or SpO_2_/FiO_2_ ≤ 315 if PaO_2_ is not available.						

### Tocilizumab (TCZ)

Studies have shown that TCZ can be used to treat patients with severe COVID-19 (Michot *et al*. 2020; Morena *et al*. 2020; Xiaoling *et al*. 2020). TCZ is a humanized monoclonal antibody against human IL-6R. TCZ specifically binds to sIL-6R or mIL-6R to inhibit downstream signaling. TCZ has been approved by the US Food and Drug Administration (FDA) and Japan Pharmaceuticals and Medical Devices Agency (PMDA) for treatment of rheumatoid arthritis (RA) (Navarro *et al*. 2014; Tanaka 2015). In addition, TCZ can also be used to treat systemic juvenile idiopathic arthritis (JIA), giant cell arteritis, and CRS (Ortiz-Martinez 2020).

It was approved by FDA in August 2017 for the standard treatment of CRS caused by chimeric antigen receptor (CAR)-genetically modified T cells (CAR-T). Intravenous injection of TCZ has been used for the treatment of CAR-T-induced severe cytokine storms (Toniati *et al*. 2020). Clinical trials are being conducted in China and Italy for COVID-19 patients who had lung tissue damages (Fundacion SEIMC-GESIDA 2020; University of Chicago 2020a, b; Klopfenstein *et al*. 2020; Malaya 2020; National Cancer Institute 2020; Roche 2020). In fact, some patients returned to normal and respiratory function based on oxygen intake and lung opacities improved remarkably (Fundacion SEIMC-GESIDA 2020; Hashizume 2020), with others experiencing returned C-reactive protein (CRP) levels and lymphocytes levels (Le *et al*. 2018).

From March 9 to 20, 2020, Italian researchers conducted a prospective study of 100 patients in the Spedali Civili of Brescia, a large university hospital at Brescia, Italy (Toniati *et al*. 2020). Confirmed COVID-19 pneumonia and ARDS patients under respiratory support were recruited and analyzed to determine whether intravenous TCZ was needed. Two consecutive intravenous infusions were performed at 12-hour intervals with a dose of 8 mg/kg TCZ. Based on the clinical outcome, the third infusion was optional. Based on the Brescia COVID respiratory severity score (BCRSS), which is zero to eight with higher score indicating higher severity, the symptom of acute respiratory failure was eased 24–72 h and 10 days following the use of TCZ (Sciascia *et al*. 2020). Apparently, TCZ effectively improved clinical symptoms and inhibited deterioration of critically ill patients (Kewan *et al*. 2020).

In December 2020, low dose TCZ, *i.e*. lower than EMA- and FDA-labeled dose (8 mg/kg) and the emerging standard of care dose (400 mg), was tested in 332 patients with COVID-19 pneumonia. This study aimed to clarify whether lower dose is effective, as well as reducing the risk of secondary bacterial infections and extension of its limited supply (University of Chicago 2020a).

This result concurred with another clinical trial, which differentiated non-survivors from survivors based on serum IL‐6 levels (Quartuccio *et al*. 2020). Another study of 32 patients in the United States proposed low-dose TCZ treatment. Fever was relieved (75.0% vs. 34.2%, *p* = 0.001) and CRP was decreased (86.2% vs. 14.3%, *p* < 0.001) within the 24–48 hours following TCZ administration. In addition, fever resolution or CRP decline seemed irrelevant to TCZ doses, with *p* equal to 0.80 and 0.10, respectively. The median time to clinical recovery was three days (IQR, 2–5) for survived patients. Five (15.6%) patients died within the follow-up of 28 days. (Strohbehn *et al*. 2020).

Evidences indicated that patients with diabetics or hyperglycemia (non-diabetics) couldn’t get optimal control through TCZ. Use of dose followed the guidance of the treatment dosage for systemic juvenile idiopathic arthritis (sJIA), *i.e*. regularly 8 mg/kg (12 mg/kg for weight <30 kg), once every two weeks. After receiving one to two doses of TCZ, about 70% of patients induced the immune response within 14 days (average four days) (Chen 2020; Yokota *et al*. 2008). TCZ improved blood oxygen saturation, abnormal computed tomography and the number of lymphocytes, and normalized C-reactive protein (CRP) levels in most patients (Campochiaro *et al*. 2020). TCZ inhibits IL-6 in the immune response and changes the way how a patient’s immune system works, which may make patients more susceptible to infection or worsen the current infection, and even cause death due to deterioration of disease. Some studies had showed that the main issue with TCZ treatment was serious infection. In one clinical study in Italy, four patients (13%) developed bacterial infection after receiving TCZ treatment (Pawar *et al*. 2020). Results showed that common side effects observed in patients receiving TCZ, include upper respiratory tract infections, headaches and high blood pressure, *etc*. Severe side effects resulted from long-term use include: damaged tissues in stomach or intestine, liver disease (hepatotoxicity), hypogammaglobulinemia (Vilchez-Oya *et al*. 2020), reduced and low platelet count (Jones *et al*. 2010) and elevated blood cholesterol levels. Moreover, it increased the risk of cancers, hepatitis B infection and severe allergic reactions, neurological problems and death (Aeschlimann *et al*. 2020). Still, wider and comprehensive clinical trials are needed to evaluate safety and effectiveness of TCZ in COVID-19.

### Sarilumab

Sarilumab is a monoclonal antibody, specifically blocking the action of IL-6 (Pharmaceuticals 2020). Sarilumab binds to sIL-6R, therefore preventing IL-6 from binding to its receptor and stopping IL-6 signaling (Atzeni *et al*. 2019). Affinity of sarilumab to human IL-6R is stronger than TCZ, and its half-life is extended. With standard clinical doses, the overall efficacy and safety of sarilumab seemed comparable to TCZ (Ogata *et al*. 2019). Sarilumab was approved as a fully humanized monoclonal antibody against IL-6R, first in 2017 in Japan and then in the United States and the European Union for the treatment of rheumatoid arthritis (Boyce *et al*. 2018).

Studies have shown that sarilumab are promising for treating patients with moderate COVID-19 infection (Clinica Universidad de Navarra 2020; Pharmaceuticals 2020; Pinedo 2020; Sacco 2020; Westyn Branch-Elliman 2020). In one clinical trial with 120 moderate patients infected with SARS-CoV-2, subcutaneous injection of sarilumab (400 mg) was performed, using intubation or death within 14 days as the primary endpoint. Anti-viral medicines, such as remdesivir or hydroxychloroquine, were optional (Westyn Branch-Elliman 2020). In another clinical trial with sarilumab (175 mg/mL in 1.14 mL solution) added on patients’ standard daily therapy after 24 hours from hospitalization, which was followed by the dosage of 200 mg subsequently after 48 and 96 hours intravenously, outcome showed that seven patients got improved on the Horovitz index and reduced in the echo score (MD *et al*. 2020; Soldati *et al*. 2020). Respiratory function was used as the primary endpoint, and evaluation of IL‐6, CRP, serum amyloid A (SAA), D‐dimer, lactate dehydrogenase, and lymphocyte count was applied as the second endpoint. It indicated that sarilumab can improve recovery from COVID-19. However, sarilumab has a higher risk for neutropenia than TCZ, but a lower risk for dyslipidemia, injection site reactions and gastrointestinal perforation (Benucci *et al*. 2020). Further clinical trials are still needed on sarilumab in COVID-19 to evaluate its safety and efficacy.

### Siltuximab

Siltuximab (CNTO 328, Sylvant) is a human-mouse chimeric monoclonal antibody (MAb) (LactMed 2019) against IL-6. It is an effective IL-6 inhibitor, which can prevent IL-6 from binding to sIL-6R. Siltuximab interferes with IL-6-mediated B lymphocyte and plasma cell growth, vascular endothelial growth factor (VEGF) secretion, and autoimmunity (Fajgenbaum and Kurzrock 2016). Siltuximab received FDA approval in 2014 for the treatment of patients with multicenter Castleman disease (Chen *et al*. 2016). Siltuximab may be a viable monoclonal antibody for COVID-19, but studies have suggested that siltuximab should not be used in patients with CRS (Wang *et al*. 2004).

One clinical trial in Italy focused on consecutive patients, who confirmed by interstitial pneumonia and positive test for SARS-COV-2, were recruited for the study of treatment with siltuximab. These patients had developed serious respiratory complications, defined by the need of ventilation (either invasive or non-invasive). Retrospective data were collected with the study completed in June 2020, but the results have not been released yet (Giuseppe Gritti 2020). Current clinical trials on effectiveness and safety of siltuximab in the treatment of COVID-19 are still in progress (Giuseppe Gritti 2020; Judit Pich Martínez 2020). Whether siltuximab is suitable for the treatment of COVID-19 still needs further studies.

### Olokizumab

Olokizumab (CDP6038) is a humanized anti-IL-6 monoclonal antibody, which aims at IL-6 instead of its receptor and selectively blocks the final assembly of the signaling complex. In studies with rheumatoid arthritis, olokizumab showed potential to relieve rheumatoid arthritis symptoms, with a median plasma half-life of ~31 days, 63% bioavailability and no apparent antidrug-antibody-mediated clearance (Genovese *et al*. 2014). However, no enough clinical trials of olokizumab have been released (R-Pharm International *et al*. 2020). Therefore, there is still a long way for clarify whether olokizumab is appropriate for COVID-19 therapy.

## CONCLUSION

Since the outbreak of COVID-19 in December 2019, within a few months, more than seven million people have been infected worldwide. COVID-19 has higher morbidity and relatively higher lethality. Clinical studies of symptoms and virology researches have showed that deterioration of patients' condition was largely due to the severity of cytokine storm in human body. Cytokine storms were initially generated in patients' lungs, and then spread to the whole body, which eventually led to patients' systemic organ failure and ultimate death. Cytokine IL-6 plays a vital role to induce a cytokine storm. Inhibiting the pro-inflammatory effect of IL-6 will be an important method to alleviate cytokine storms in patients. Searching for monoclonal antibodies of IL-6 or IL-6R will be critical for development of COVID-19 therapeutic drugs. At the same time, valuable clues for the treatment of COVID-19 can probably be found via going through application of IL-6 blockers in rheumatoid diseases and JIA, which are more mature fields in IL-6 inhibitors. Potential anti-inflammatory monoclonal antibodies against IL-6 currently proposed include TCZ, sarilumab and siltuximab. Among them, research on TCZ is more comprehensive. It is also widely regarded as an effective drug for the treatment of COVID-19. Now there is still much debate about application of sarilumab, siltuximab and olokizumab in therapy. Whether they can effectively treat COVID-19 still needs further investigation.

## Conflict of interest

Jingrui Jiang, Jun Wang, Lulu Yao, Shenghan Lai and Xueji Zhang declare that they have no conflict of interest.
